# Molecular characterization and gene expression data of liver expressed antimicrobial Peptide-2 (LEAP-2) isolated from rock bream (*Oplegnathus fasciatus*)

**DOI:** 10.1016/j.dib.2019.104538

**Published:** 2019-09-19

**Authors:** Seong Don Hwang, Min Soo Joo, Jee Youn Hwang, Mun-Gyeong Kwon, Ji-Min Jeong, Jung Soo Seo, Bo-Yeong Jee, Chan-Il Park

**Affiliations:** a Aquatic Animal Disease Control Center, National Institute of Fisheries Science (NIFS), 216 Gijanghaean-ro, Gijang-eup, Gijang-gun, Busan 46083, Republic of Korea; b Institute of Marine Industry, College of Marine Science, Gyeongsang National University, 455, Tongyeong 650-160, Republic of Korea

**Keywords:** Rock bream, Liver expressed antimicrobial Peptide-2, Innate immune system, Antimicrobial peptides, Antimicrobial activity

## Abstract

Antimicrobial peptides (AMPs) are known to play a role as a first line of defence against microbial invasion. Liver Expressed Antimicrobial Peptides-2 (LEAP-2) is one of the AMPs. LEAP-2 includes four highly conserved cysteine residues and belongs to a cysteine-rich peptides group. We identified and characterized the molecular properties of LEAP-2 in rock bream. The expression levels of rock bream LEAP-2 (RbLEAP-2) in the 12 different tissues of healthy fish and the RbLEAP-2 expression pattern after infections with *Edwardsiella piscicida* (*E. piscicida*), *Streptococcus iniae* (*S. iniae*) and red seabream iridovirus (RSIV) were examined. This data provide that RbLEAP-2 plays an important role in innate immunity when rock bream is infected with a pathogen.

**Table:** Specifications Table

Subject area	Immunology and Microbiology
More specific subject area	Immunology
Type of data	Table and figure
How data was acquired	Using various program, for example, Next Generation Sequencing (NGS), BLASTX from the National Center for Biotechnology Information (NCBI), GENETYX ver. 8.0, PotParam tool on the ExPASy Proteomics Server, Simple Modular Architecture Research Tool (SMART), ClustalW, Mega 4, and I-Tasser server to confirm the molecular characteristics of RbLEAP-2. Total RNA was extracted from various tissues of rock bream infected
Data format	Raw and analysed
Experimental factors	RbLEAP-2 Molecular characterizations were confirmed and RbLEAP-2 gene expression profiles were compared between healthy controls and groups with bacterial and viral infections.
Experimental features	The sequence of the RbLEAP-2 gene was identified by NGS analysis, and molecular and expression characteristics were confirmed.
Data source location	Gyeongsang National University, Tongyeong, Republic of Korea
Data accessibility	The data are available with this article
Related research article	A. Krause, R. Sillard, B. Kleemeier, E. Kluver, E. Maronde, J. R. Conejo-Garcia, W. G. Forssmann, P. Schulz-Knappe, M. C. Nehls, F. Wattler, S. Wattler, K. Adermann, Isolation and biochemical characterization of LEAP-2, a novel blood peptide expressed in the liver. Protein Sci. 12 (2003) 143–52.

**Table:** 

**Value of the Data** • These data provide information on the antimicrobial peptides that have antimicrobial activity directly on the pathogen, among innate immunity, an important immune system of rock bream. • These data provide the expression patterns of RbLEAP-2 for immune responses in rock bream infected with various pathogens, and will be fundamental to improving the understanding of the role of RbLEAP-2. • Based on these tissue-specific expression data sets, further studies on related genes will contribute to gaining insights into the limited functions of LEAP-2 in teleost. • The antimicrobial activity data can be an important basic data for developing new antimicrobial products.

## 1 Data

**Fig. 1:**
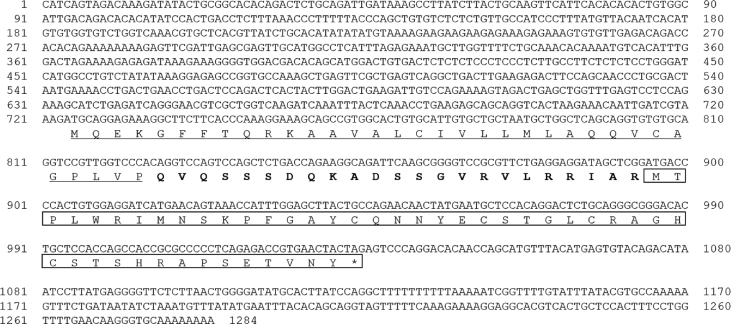
cDNA and deduced amino acid sequences of RbLEAP-2. The signal peptide is underlined, a prodomain is in bold letters and the mature peptide domain is indicated with a box.

**Fig. 2:**
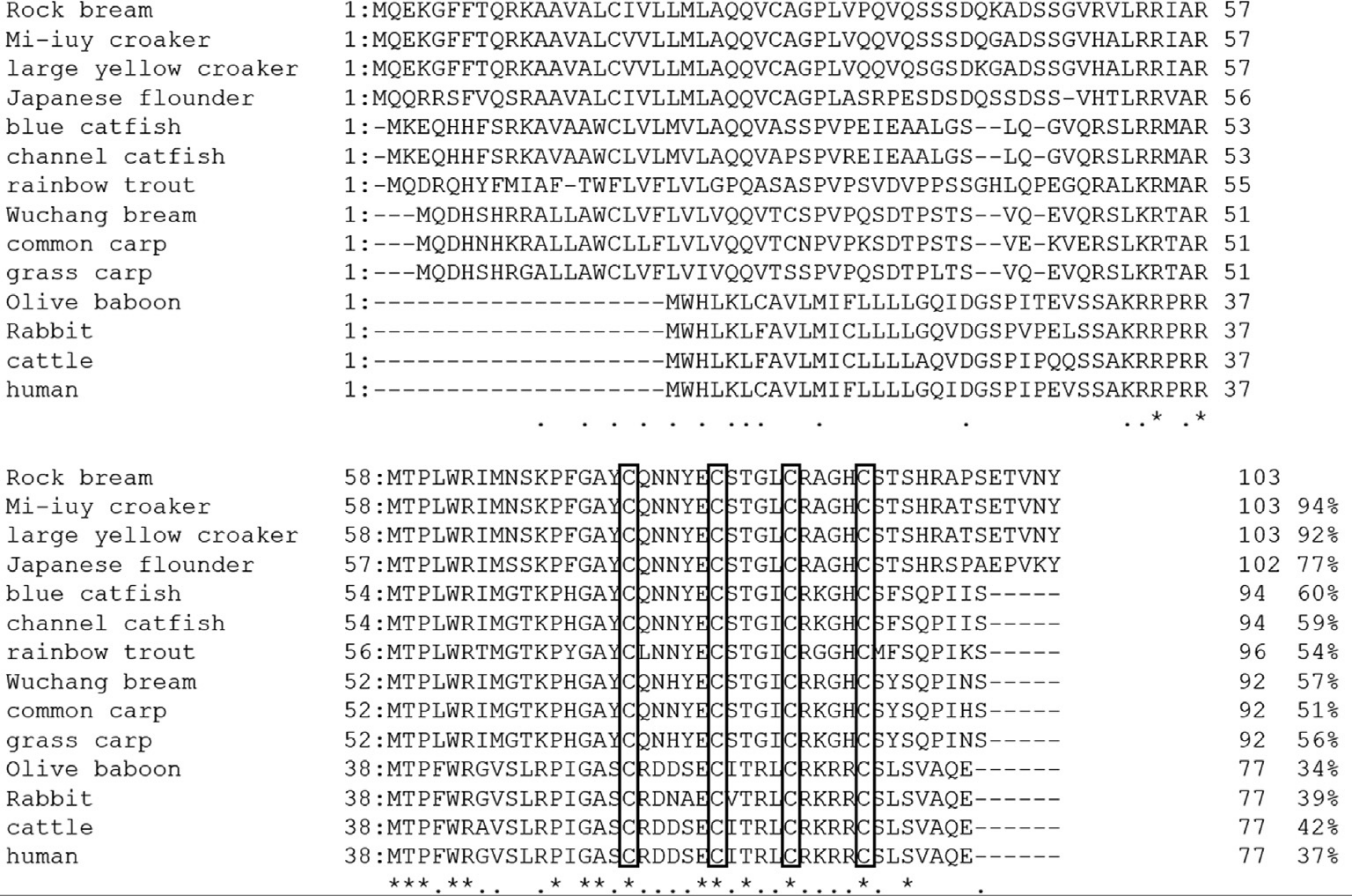
Multiple alignments of RbLEAP-2 with the LEAP-2 amino acid sequences of other animals. NCBI accession numbers of LEAP-2 are as follows: Blue catfish, AY845142.1; Cattle, CAC51470; Channel catfish, NM_001200205.1; Common carp, KC551971.1; Grass carp, FJ390414.1; NM_153069.3; Human, NM_052971.2; Japanese flounder, EU586111.1; Large yellow croaker, JN991058.1; Mi-iuy croaker, KJ000088.1; Olive baboon, NP_001162235; Rabbit, NP_001164729; Rainbow trout, NP_001117936; and Wuchang bream, JQ344324.1.

**Fig. 3:**
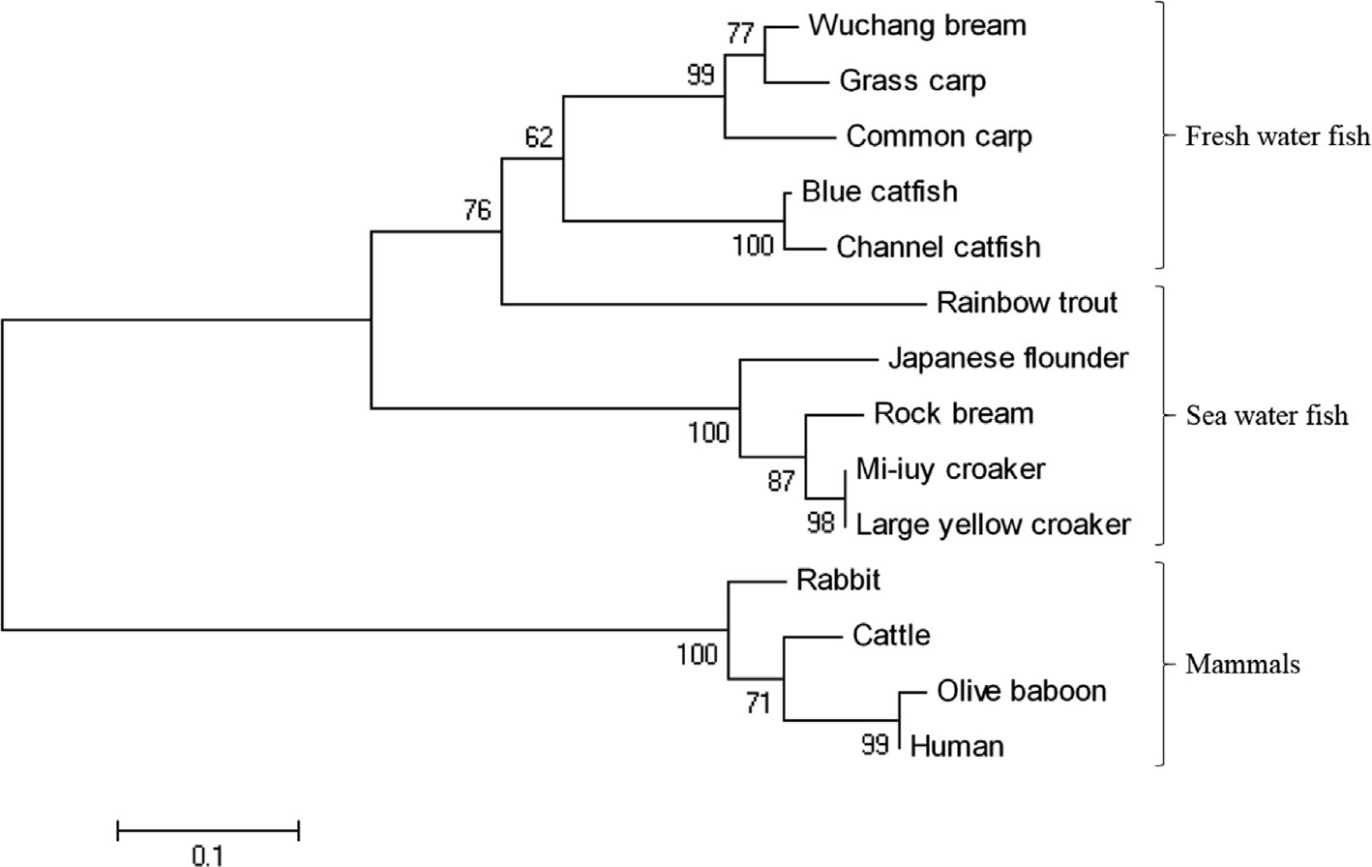
Phylogenetic analysis of the deduced LEAP-2 amino acid sequences in various animals. The phylogenetic tree was constructed using the neighbour-joining method using the MEGA 4 software. Bootstrap sampling was performed with 2000 replicates. The scale bar is equal to 0.1 change per amino acid position.

**Fig. 4:**
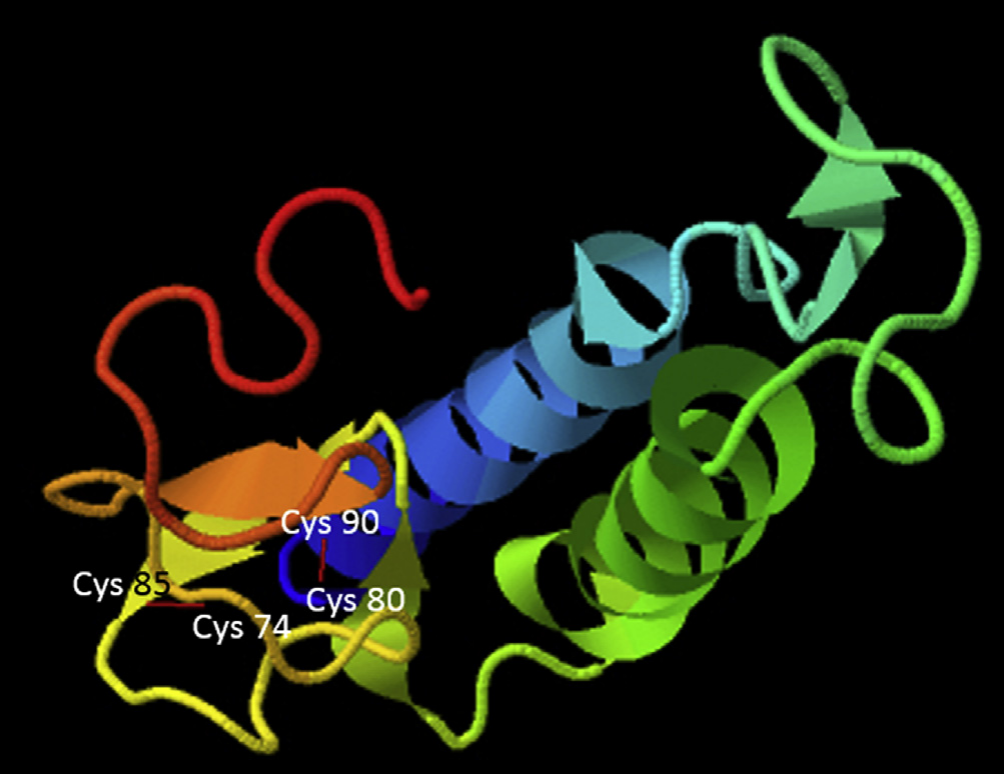
Three-dimensional structure of RbLEAP-2. The highly conserved cysteine residues, disulphide bonds (Red lines), α-helical and β-sheet structure are shown.

**Fig. 5:**
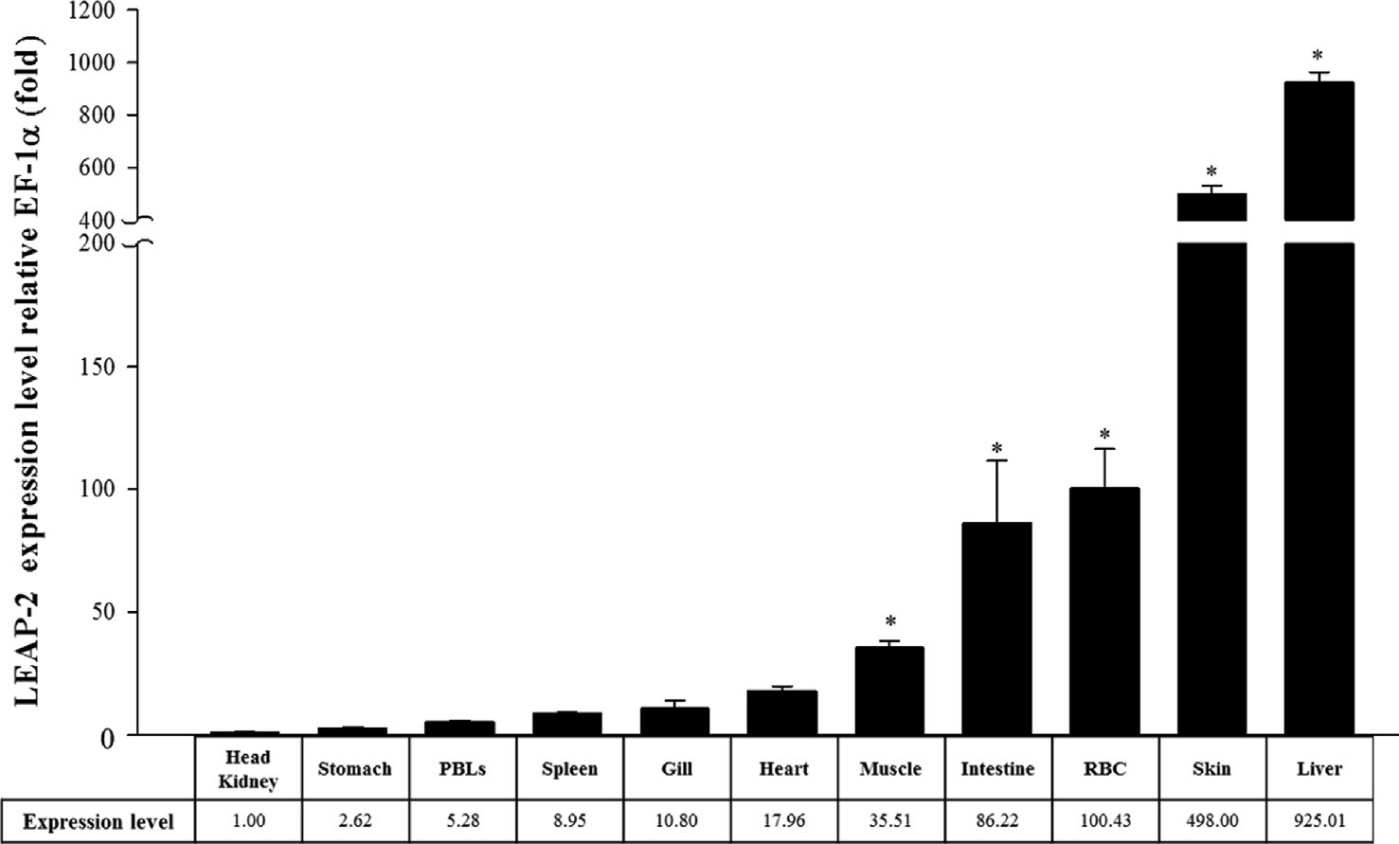
Detection of RbLEAP-2 genes in different tissues of normal rock breams using real-time PCR. The reference gene, EF-1α was used for normalizing the real-time PCR data. Data are presented as the mean ± SD from three independent cDNA samples and three replicates from each sample. Asterisks indicate significant differences (*p* < 0.05) compared to the head kidney samples.

**Fig. 6:**
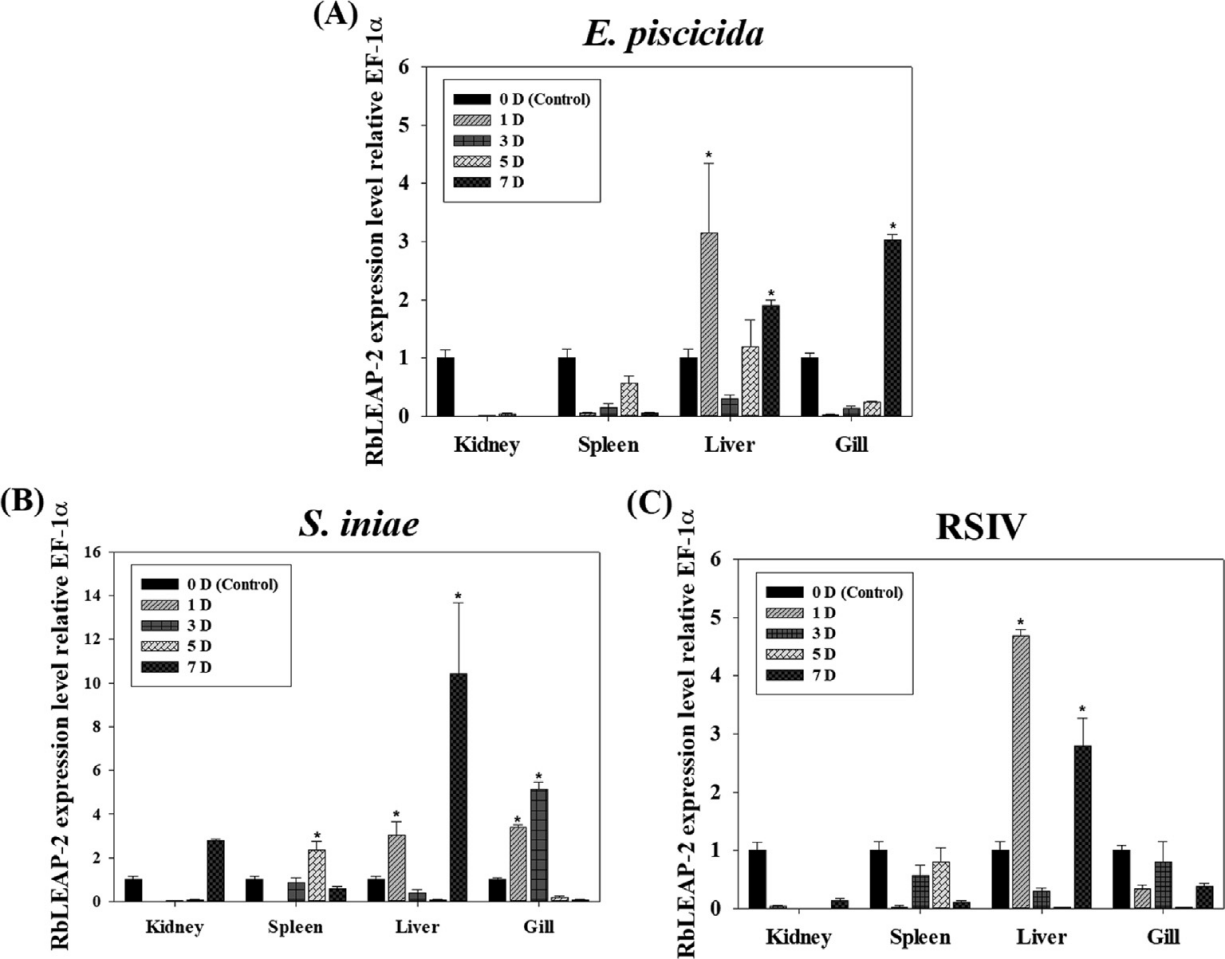
Gene expression levels of RbLEAP-2 in the kidney, spleen, liver and gills after infections with *E. piscicida* (A), *S. iniae* (B), and RSIV (C). Levels of RbLEAP-2 transcripts were quantified relative to those of EF-1α levels. Data are presented as the mean ± SD from three independent cDNA samples and three replicates for each sample. Asterisks represent significant differences compared to the control (0 h) group as analysed using ANOVA (*p* < 0.05).

AMPs are known to play an important role as a first line of defence against microbial invasion within the innate immune system [1]. LEAP-2 contain four and two pairs of disulphide bonds, respectively, and belong to a cysteine-rich peptides group, which is one of the five major groups of AMPs [2]. In a previous study, LEAP-2 was identified to be synthesised in the liver, secreted into the blood and eliminated by the kidney [2]. The full-length cDNA sequence (1284 bp) of RbLEAP-2 (GenBank accession No. MF464024) contains an open reading frame (ORF) of 309 bp that encodes 103 amino acid (aa) residues, the 5′-untranslated region (UTR) of 723 bp and the 3′-UTR of 249 bp. The predicted molecular weight of RbLEAP-2 is 11.37 kDa and its p*I* is 9.45. A predicted signal peptide region (1–29 aa), prodomain region (30–57 aa) and mature peptide region (58–93 aa) are conserved in RbLEAP-2 (Fig. 1). Multiple alignments of RbLEAP-2 with other known fish and mammal LEAP-2s determined the sequences with the highest identity as Mi-iuy croaker LEAP-2 (94%), and the lowest identities as Japanese eel LEAP-2 in fish (58%) and Human LEAP-2 in mammals (37%) (Fig. 2). LEAP-2s of all species, including the RbLEAP-2, have a highly conserved set of four cysteines, which form two disulphide bonds. There is a conserved sequence “RXXR” motif located at the cleavage site between the prodomain region and the mature peptide region in the all tested fish species. In addition, there is a conserved sequence “RXGH” motif in fish, and the “RKRR” motif in mammals in the mature peptide regions. Conserved motifs composed of six amino acids “MTPLWR” and “MTPFWR” are found in fish and mammals, respectively, at the beginning of the mature peptide. Phylogenetic analysis was performed using the amino acid sequences of other known fish LEAP-2s. The overall topology of the phylogenetic tree was divided into three groups of freshwater fish, seawater fish and mammals (Fig. 3). The RbLEAP-2 showed the closest similarity to the Mi-iuy croaker and the large yellow croaker. The putative structure of RbLEAP-2 was modelled using I-Tasser (Fig. 4). The I-Tasser data indicated that the TM-score of the model was 0.55 ± 0.15, the C-score was −1.62, with an estimated RMSD of 6.7 ± 4.0 Å. In the three-dimensional structure, the pairs (Cys74 and Cys85), and (Cys80 and Cys90) form disulphide bonds. RbLEAP-2 was predicted to form an amphipathic α-helical structure and β-sheet structure (Fig. 4). The RbLEAP-2 expression levels in the tissues of normal rock bream were shown in the graph and were compared to those of the head kidney, which showed the lowest expression levels. RbLEAP-2 showed the highest expression levels (925.01 fold) in the liver, skin (498.00 fold), RBC (100.43 fold), intestine (86.22 fold) and muscle (35.51 fold) (Fig. 5). Following the bacterial and viral infections, the expression patterns of the RbLEAP-2 gene in the kidney, spleen, liver and gill samples were analysed by qRT-PCR. In case of fish infected with *E. piscicida*, significant expression of RbLEAP-2 was observed in the liver (3.14 fold at 1 day and 1.9 fold at 7 days after challenge) and gills (3.02 fold at 7 days after challenge), but the expression levels were low in the kidney and spleen. In the case of the fish infected with *S. iniae*, significant up-regulation of RbLEAP-2 levels in the spleen (2.34 fold at 3 days after challenge), liver (3.03 fold at 1 day and 10.43 fold 7 days after challenge) and gills (3.41 fold at 1 day and 5.15 fold at 5 days after challenge) were observed. The expression of RbLEAP-2 was significantly increased only in the liver of RSIV-infected fish (4.67 fold at 1 day and 2.79 fold at 7 days after challenge) (Fig. 6). The minimal inhibitory concentration (MIC) of RbLEAP-2 against *E. coli, S. iniae, Vibrio alginolyticus and V. campbellii* was 312.5–625 μg/mL. RbLEAP-2 showed MIC against *E. piscicida* at 2500 μg/mL (Table 2).

**Table 1:** PCR primers used in this study.

Target	Usage	Primer sequence (5′–3′)
RbLEAP-2	Real-time F	GAGGAGGATAGCTCGGATGA
	Real-time R	CAGAGTCCTGTGGAGCATTC
EF-1α	Real-time F	CCCCTGCAGGACGTCTACAA
	Real-time R	AACACGACCGACGGGTACA

**Table 2:** Antimicrobial activity of RbLEAP-2.

Bacterial strain	Gram	°C	MIC (μg/ml)
*Escherichia coli* (JM109)	(−)	37	312.5–625
*Edwardsiella piscicida* (ET883)	(−)	27	>2500
*Streptococcus iniae* (FP5228)	(+)	27	312.5–625
*Vibrio alginolyticus* (KCCM2928)	(−)	27	312.5–625
*V. campbellii* (KCCM40684)	(−)	27	312.5–625

## 2 Experimental design, materials, and methods

### 2.1 Molecular characterization of RbLEAP-2

To obtain the full-length of RbLEAP-2 cDNA sequence, Next Generation Sequencing (NGS) analysis was performed on rock bream liver stimulated with *Streptococcus iniae* and Red Seabream Iridovirus (RSIV). The isolated base sequence of the full-length RbLEAP-2 cDNA and the corresponding amino acid sequences were determined using the BLASTX programme (http://blast.ncbi.nlm.nih.gov/Blast.cgi) from the National Center for Biotechnology Information (NCBI) and GENETYX ver. 8.0 (SDC Software Development, Japan). The molecular weight and isoelectric points (p*I*) were determined using the ProtParam tool on the ExPASy Proteomics Server (http://web.expasy.org/protparam/). To identify the characteristic domains, Simple Modular Architecture Research Tool (SMART) (http://smart.embl-heidelberg.de/) was used. Multiple sequence alignments were analysed using ClustalW (http://www.genome.jp/tools/clustalw/). The phylogenetic analysis was inferred using the Mega 4 programme and the distance analysis was conducted using the neighbour-joining method [3]. Bootstrap sampling was performed with 2000 replicates. The tertiary structure prediction was performed using the I-Tasser server (http://zhang.bioinformatics.ku.edu/I-TASSER/about.html) [4], [5].

### 2.2 Fish

Rock breams were obtained from the Gyeongsangnam-do Fisheries Resources Research Institute, Korea. The weights and body lengths of these fish were 68.5 ± 10 g and 14.3 ± 1 cm, respectively. The fish were kept in tanks with filtered running water at 20–23 °C and acclimatized for 2 weeks before the experiments. The fish were fed with commercial feed once a day.

### 2.3 RbLEAP-2 gene expression analysis in normal fish

Three normal fish were anesthetized with Benzocaine (Sigma-Aldrich, USA) to obtain blood cells and tissues samples. To obtain the peripheral blood leukocytes (PBLs) and red blood cells (RBCs), percoll density gradient method was used (Sigma-Aldrich) as described previously [6]. The peripheral blood samples were collected from caudal veins with heparin-treated syringes. The collected peripheral blood samples were separated by adding them to RPMI1640 (Invitrogen, USA) and 53% Percoll density gradients (Sigma-Aldrich). Five mL of percoll solution was mixed with percoll: 10 × phosphate-buffered saline (PBS): 1 × PBS at a ratio of 53: 5.9: 41.1 and 5 mL of blood was suspended in RPMI1640 and dispensed into the layers and centrifuged at 400 g for 20 minutes. The separated PBLs and RBCs were washed three times with 1 × PBS, and the pellets obtained by centrifugation were used for the experiments. The sampled fish were euthanized and aseptically anatomized, and we collected the head kidney, spleen, liver, intestine, gill, muscle, heart, skin, and stomach tissues. TRIzol reagent (Invitrogen) was used to extract total RNA, and first-strand cDNA synthesis was conducted using a first-strand cDNA synthesis kit (Roche, Germany) according to the manufacturer's instructions. Quantitative real-time PCR (qRT-PCR) was performed with SYBR Green Master Mix (Takara, Japan) following the manufacturer's protocol. qRT-PCR was conducted with cDNA templates of each tissue and the specific primer sets for RbLEAP-2 was designed using Primer3 ver. 0.4.0 (http://bioinfo.ut.ee/primer3-0/4/0/) based on the full-length cDNA sequences of RbLEAP-2 (Table 1). Amplification was performed by initial denaturation at 50 °C for 4 min and 95 °C for 10 min followed by 45 cycles at 95 °C for 20 sec and 60 °C for 1 min with a final dissociation at 95 °C for 15 sec, 60 °C for 30 sec and 95 °C for 15 sec. The expression levels of the RbLEAP-2 gene were determined by the 2^–ΔΔCT^ method using EF-1α expression as the reference gene [7]. All data were reported as levels of RbLEAP-2 mRNA relative to EF-1α mRNA and expressed as mean ± standard deviation (SD). The significance of the differences in gene expressions among the tissues were determined by one-way analysis of variance (ANOVA) in Predictive Analytics Software statistics ver. 18.0 (PASWStatistics 18) and were considered significant when *p* < 0.05. Multiple comparisons were performed using the Tukey HSD test.

### 2.4 RbLEAP-2 gene expression analysis after pathogen infections

To analyse the immune response of RbLEAP-2 against various pathogens, normal rock bream were challenged by intraperitoneal injection with *E. piscicida*, *S. iniae* or RSIV suspended in phosphate-buffered saline (PBS). The pathogens were adjusted to 1.5 × 10^5^, 1.5 × 10^5^ cells/fish and 1 × 10^4^ copies/fish, respectively, and the challenged fish were kept in seawater at 23–26 °C. Liver, kidney, spleen and gill samples were taken from randomly selected three fish immediately after infection at 0, 1, 3, 5 and 7 days post-infection for experimental groups. Total RNA extraction, cDNA synthesis and qRT-PCR were conducted as described above (section 2.3.). The significance of the differences in gene expression levels between the pathogen-infected and control groups were determined by ANOVA (*p* < 0.05). Multiple comparisons were performed using the Tukey HSD test (*p* < 0.05).

### 2.5 Antimicrobial activity assay

To analyse the antimicrobial activities against various bacteria, the RbLEAP-2 peptides were synthesised to over 90% purity (GL Biochem, Shanghai, China) based on the amino acid sequences of the mature peptide containing two disulphide bonds (Cys74 with Cys85, Cys80 with Cys90). The bacteria shown in Table 2 were used. Each bacterial strain was cultured on brain heart infusion agar (BHIA) plates. The minimal inhibitory concentration (MIC) was determined by the broth microdilution method with some modification [8]. The synthetic peptide was dissolved in 0.5% Dimethyl Sulphoxide (DMSO), and diluted stepwise in 2 fold volumes with Mueller-Hinton (MH) broth in a 96 well plate. 50 μL aliquots of the synthetic peptides at various concentrations and 50 μL of each strain diluted to 5 × 10^5^ cfu/mL were mixed in a 96 well plate and cultured for 12 hours with shaking at 27 °C. The 0.5% DMSO solvent was diluted stepwise with MH broth, mixed with the bacteria and cultured, which was designated the control group. For accuracy, this experiment was repeated three times. After incubation, the Optical Density (OD) was measured at 540 nm using a VICTOR^3^ 1420 Multilabel Counter (PerkinElmer, Waltham, MA, USA). The MIC range was set to the lowest concentration of the peptides at which the growth of the bacteria was inhibited.

## Appendix A Supplementary data

Supplementary Fig. 1 Analysis of RbLEAP-2 gene expression using quantitative real-time PCR products in various tissues after infection. The products were detected on agarose gel by electrophoresis. Lanes M-10: (M) marker. Expression in liver on day 1 (1), liver on day 7 (2) and gill on day 7 (3) after *E. piscicida* infection. Expression in spleen on day 5 (4), liver on day 1 (5), liver on day 7 (6), gill on day 1 (7) and gill on day 3 (8) after *S. iniae* infection. Expression in liver on day 1 (9) and liver on day 7 (10) after RSIV infection. (a): EF-1α (46 bp), (b): RbLEAP-2 (99 bp). Supplementary Fig. 1
