# Supplementary figures and images for: Molecular characterization and gene expression data of liver expressed antimicrobial Peptide-2 (LEAP-2) isolated from rock bream (Oplegnathus fasciatus)
Source: Data Brief. 2019 Sep 19;26:104538. doi: 10.1016/j.dib.2019.104538 (PMC6811885; doi:10.1016/j.dib.2019.104538)

## Slide 1
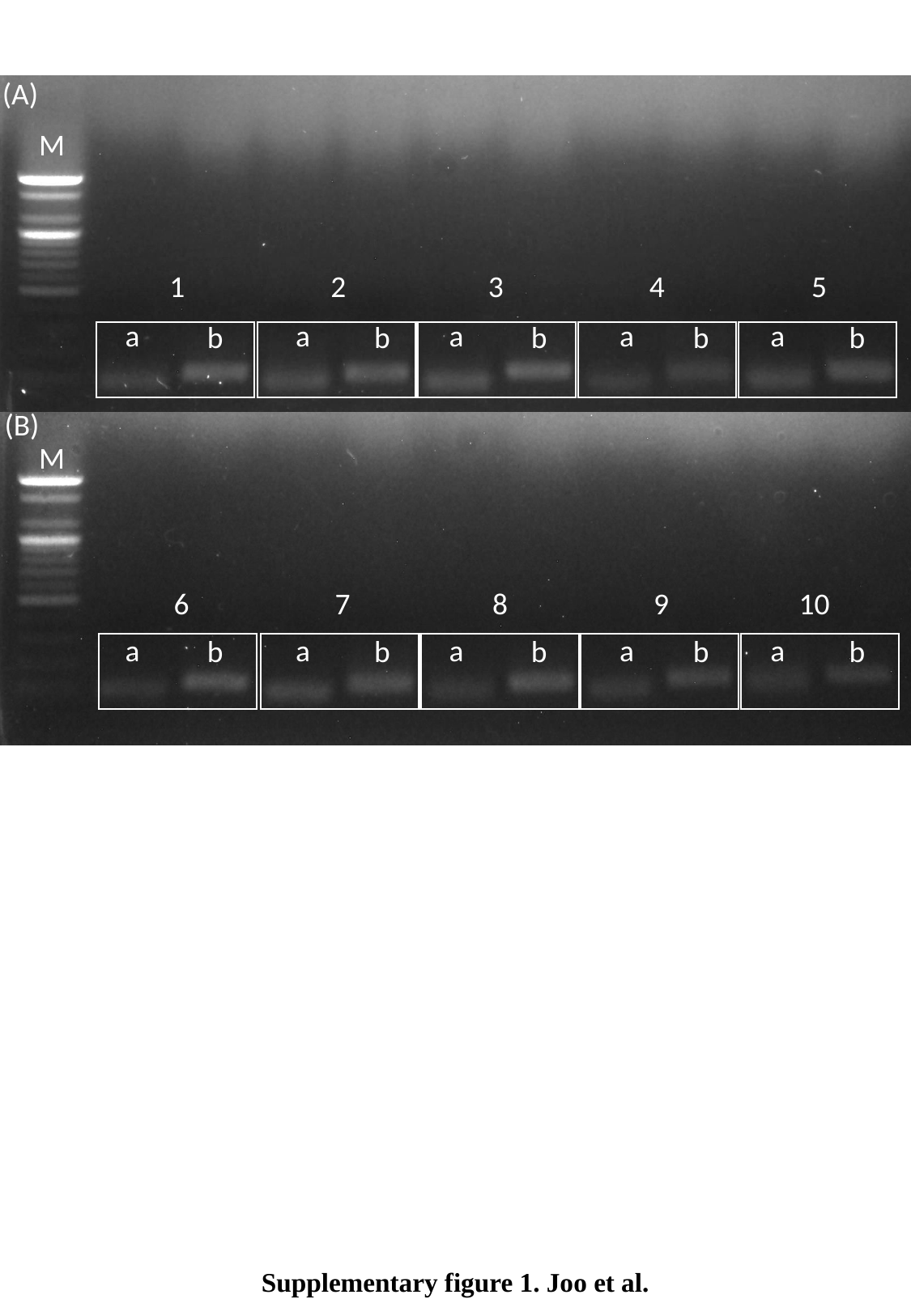

(A)
M
1
2
3
4
5
a
a
a
a
a
b
b
b
b
b
(B)
M
6
7
8
9
10
a
a
a
a
a
b
b
b
b
b
Supplementary figure 1. Joo et al.

Supplement: Supplementary Fig. 1 — Analysis of RbLEAP-2 gene expression using quantitative real-time PCR products in various tissues after infection. The products were detected on agarose gel by electrophoresis. Lanes M-10: (M) marker. Expression in liver on day 1 (1), liver on day 7 (2) and gill on day 7 (3) after E. piscicida infection. Expression in spleen on day 5 (4), liver on day 1 (5), liver on day 7 (6), gill on day 1 (7) and gill on day 3 (8) after S. iniae infection. Expression in liver on day 1 (9) and liver on day 7 (10) after RSIV infection. (a): EF-1α (46 bp), (b): RbLEAP-2 (99 bp). [file mmc1.pptx]
